# Identification of high likelihood of dementia in population-based surveys using unsupervised clustering: a longitudinal analysis

**DOI:** 10.1186/s13195-023-01357-9

**Published:** 2023-11-29

**Authors:** Amin Gharbi-Meliani, François Husson, Henri Vandendriessche, Eleonore Bayen, Kristine Yaffe, Anne-Catherine Bachoud-Lévi, Laurent Cleret de Langavant

**Affiliations:** 1 Neuropsychologie Interventionnelle, U955 E01, Institut Mondor de Recherche Biomédicale & Département d’études Cognitives, INSERM, Ecole Normale Supérieure, Université PSL, Université Paris-Est Créteil, Creteil, 94000 France; 2 Institut Agro, Univ Rennes1, CNRS, IRMAR, Rennes, 35000 France; 3 Laboratoire de Neurosciences Cognitives et Computationnelles, Département d’études Cognitives, Ecole Normale Supérieure, Université PSL, INSERM, Paris, 75005 France; 4 Département de Rééducation Neurologique, Sorbonne Université, Hôpital Pitié-Salpêtrière–Assistance Publique Hôpitaux de Paris, Paris, 75013 France; 5 Global Brain Health Institute, University of California, San Francisco, CA 94143 USA; 6 Departments of Psychiatry, Neurology and Epidemiology and Biostatistics, University of California, San Francisco, CA 94143 USA; 7 Service de Neurologie, Centre de référence maladie de Huntington, Hôpital Henri Mondor, Assistance Publique Hôpitaux de Paris, 1 rue Gustave Eiffel, Creteil, 94000 France

**Keywords:** Dementia, Identification, Population-based surveys, Longitudinal analysis, Unsupervised clustering, Machine Learning, Multistate models

## Abstract

**Background:**

Dementia is defined as a cognitive decline that affects functional status. Longitudinal ageing surveys often lack a clinical diagnosis of dementia though measure cognition and daily function over time. We used unsupervised machine learning and longitudinal data to identify transition to probable dementia.

**Methods:**

Multiple Factor Analysis was applied to longitudinal function and cognitive data of 15,278 baseline participants (aged 50 years and more) from the Survey of Health, Ageing, and Retirement in Europe (SHARE) (waves 1, 2 and 4–7, between 2004 and 2017). Hierarchical Clustering on Principal Components discriminated three clusters at each wave. We estimated probable or “Likely Dementia” prevalence by sex and age, and assessed whether dementia risk factors increased the risk of being assigned probable dementia status using multistate models. Next, we compared the “Likely Dementia” cluster with self-reported dementia status and replicated our findings in the English Longitudinal Study of Ageing (ELSA) cohort (waves 1–9, between 2002 and 2019, 7840 participants at baseline).

**Results:**

Our algorithm identified a higher number of probable dementia cases compared with self-reported cases and showed good discriminative power across all waves (AUC ranged from 0.754 [0.722–0.787] to 0.830 [0.800–0.861]). “Likely Dementia” status was more prevalent in older people, displayed a 2:1 female/male ratio, and was associated with nine factors that increased risk of transition to dementia: low education, hearing loss, hypertension, drinking, smoking, depression, social isolation, physical inactivity, diabetes, and obesity. Results were replicated in ELSA cohort with good accuracy.

**Conclusions:**

Machine learning clustering can be used to study dementia determinants and outcomes in longitudinal population ageing surveys in which dementia clinical diagnosis is lacking.

**Supplementary Information:**

The online version contains supplementary material available at 10.1186/s13195-023-01357-9.

## Introduction

Major neurocognitive disorder (MND), commonly known as dementia, is a clinical syndrome characterised by a decline in cognitive performance that compromises patient’s independence [1]. Repeated clinical visits and assessments reveal the progression from a healthy state to dementia. International diagnostic criteria are available to identify dementia cases. Yet, more than half of the cases in high income countries (HIC) [2] and up to 90% in low and middle income countries (LMIC) [3] remain undetected. For such, new methods are needed to identify dementia cases and to study dementia determinants at the level of countries or continents.

Several population-based surveys, modelled on the United-States Health and Retirement Study (HRS), are conducted in multiple countries to study the impact of the transition from late-life work to retirement [4]. The “HRS family” studies offer the opportunity to compare ageing outcomes internationally [5]. Yet, in these and in many other surveys, clinical dementia status is either not available or only self-reported by participants or their families, which underestimates the real number of cases.

In the absence of clinical diagnosis in population ageing surveys, unsupervised machine learning, generally used to discover clusters or patterns within datasets [6], can identify probable dementia cases. In a previous work, we applied an unsupervised clustering method to cross-sectional data from HRS and Survey of Health, Ageing and Retirement in Europe (SHARE) to identify high likelihood of dementia [7] based on variables related to demographics, comorbidities, functional status, mobility, cognition, and neuropsychiatric symptoms. However, applying this clustering method to cross-sectional data did not allow us to investigate longitudinal transition from normal to impaired functional status, or to assess risk factors associated with transition to dementia status.

Herein, we built a clustering analysis for identifying transition to high likelihood of dementia in population ageing surveys using repeated measurements of cognition and functional status with a modified unsupervised machine-learning algorithm. Our objectives were to demonstrate that this method can identify probable dementia in population aging surveys where dementia is either poorly or non-diagnosed, and that this method is also efficient to study dementia risk factors. Three analyses were used to ascertain the internal validity of “Likely Dementia” status: (1) comparing “Likely Dementia” identification with self-reported dementia, (2) studying the prevalence of “Likely Dementia” status according to sex and age, (3) testing whether traditional dementia risk factors were associated with a higher risk of transition to “Likely Dementia” cluster. To demonstrate replicability, we conducted our study using SHARE survey and replicated it in the English Longitudinal Study of Ageing (ELSA).

## Material and methods

### Study design and participants

We used the harmonised dataset provided by the Gateway to Global Aging [5] of SHARE, a longitudinal panel study conducted across multiple countries in Europe and Israel [8]. This population survey takes place every two years and follows a representative sample of individuals aged 50 years or older from each participating country. The harmonised version of SHARE consisted of seven waves (the third being retrospective) conducted between 2004 and 2017. We included subjects from countries who have participated in SHARE since the first wave (i.e., Austria, Belgium, Denmark, France, Germany, Greece, Israel, Italy, The Netherlands, Spain, Sweden, and Switzerland), aged 50 years or older, with consecutive follow-ups.

### Selected variables

Variables related to cognition and function were retained in compliance with the DSM-5 criteria of MND. The selected variables are listed in the Supplementary Information (Supplementary Tables 1 and 2). Variables with more than 30% missing values were discarded and the remaining data were imputed using the imputeMFA command of the missMDA R package [9].

### Clustering

We ran Multiple Factor Analysis (MFA) followed by Hierarchical Clustering on Principal Components (HCPC) using FactoMineR R package [10] and longitudinal data from all waves at the same time. MFA is a principal component method that balances for differences in the number of active variables per domain by forming active groups (details in Appendix and Supplementary Fig. 1). For the clustering, we retained only active groups that represented participants’ function or cognition (Supplementary Tables 1 and 2). Each participant, at each wave, was assigned to one of the three possible clusters (i.e., each participant could transition from one cluster to another, from one wave to another longitudinally). The number of clusters was set at three based on previous work for identification of high likelihood of dementia [7]. First wave participants who presented impaired cognition and function were singled out in a highly probable-dementia cluster (named “Likely Dementia”). Participants classified in “Likely Dementia” cluster were permanently assigned to it (i.e., making any incident case a prevalent one).

We took into account the attrition induced by study dropout and death across waves, and applied Inverse Probability Weighting (IPW) using the ipw R package [11]. For each wave, a logistic regression model was built based on the participants’ age, sex, and country of origin characteristics collected at the previous wave. Weights were derived by inverting the product of the predicted probabilities computed by the model, and then integrated into both imputation and clustering methods.

### Self-reported diagnosis of dementia

The discrimination power of our clustering method and its ability to identify “Likely Dementia” status, compared with the self-reported dementia status, was evaluated in terms of Sensitivity, Specificity and Area Under the Curve (AUC) metrics using data collected from the second wave of SHARE.

### Effect of age, sex, and risk factors for dementia

The prevalence of “Likely Dementia” status of each wave was computed by sex and by age. Participants were divided into six age groups (under 65 years, 65–69 years, 70–74 years, 75–79 years, 80–85 years, and more than 85 years).

We examined the role of several established modifiable risk factors, identified by Livingston [12], in transitioning to “Likely Dementia” cluster: low education, hearing loss, hypertension, excessive alcohol drinking, current smoking, depression, social isolation, physical inactivity, diabetes, obesity, and air pollution. Past history of traumatic brain injury was not available in the database and could not be tested. All risk factors were measured at baseline and were imputed whenever indicated.

All ordinal risk factors variables were dichotomised. Education level was categorised as high (upper secondary and vocational training or tertiary education) or low (less than upper secondary). For hearing loss, self-reported hearing capacity was used as a proxy considering it either being normal (excellent, very good, and good) or bad (fair or poor). Moderate and vigorous physical activity were merged into being physically active (frequency: more than once per week, once per week, one to three times a month) or inactive (hardly ever or never). The remaining risk factors were treated as dichotomous as they were in the database: hypertension (ever had high blood pressure vs. never had high blood pressure), drinking (21 units or more of alcohol per week vs. less than 21 units of alcohol per week), smoking (current smoker vs. non-current smoker), depression (Centre for Epidemiologic Studies Depression [CES-D] scale score greater than or equal to five vs. CES-D scale score less than five), social isolation (participating in social activities weekly vs. non-participating in social activities weekly), diabetes (ever had diabetes vs. never had diabetes), obesity (Body Mass Index [BMI] ≥ 30 kg/m^2^ vs. BMI < 30 kg/m^2^), air pollution (living in urban area vs. living in rural area).

### Multistate models

In each wave, a participant could be classified in one of the three clusters (Cluster 1, Cluster 2 or Cluster 3; see above). Data being interval-censored, we applied multistate models using MSM package [13] to study the impact of dementia risk factors on the risk of transition to “Likely Dementia” cluster.

Age was used as the time scale by calculating it as the difference between birth date and interview date in years, and then was divided by ten, in the multistate models, to facilitate the computational process without altering the Hazard Ratios (HR) results. Sex was treated as a binary variable (male or female). All transitions were adjusted for sex, and all covariates were set at baseline. Transition towards “Likely Dementia” cluster was further adjusted for age. For each risk factor, we computed its corresponding HR.

The robustness of the multistate models was checked in two steps. First, we considered death as a competing risk and added it as an absorbing state in the models. This was investigated in SHARE where death was reported consistently. Second, we excluded early prevalent and incident dementia cases by excluding participants categorised with a likelihood of dementia at first and second waves, and ran multistate analyses again.

### Replication cohort

In order to confirm our results, we chose the harmonised version of ELSA [14] as a replication cohort. The latter is a representative longitudinal panel study of people aged 50 years and over in England, and comprises nine waves ranging from 2002 to 2019.

### Standards of reporting

We followed both STROBE (STrengthening the Reporting of OBservational studies in Epidemiology) and MELODEM (The MEthods in LOngitudinal research on DEMentia) guidelines [15, 16] for the reporting of this study.

### Role of the funding source

Sponsors of the study had no role in study design, data collection, data analysis, data interpretation, or writing of the report.

## Results

### Identification of probable dementia

**Fig. 1:**
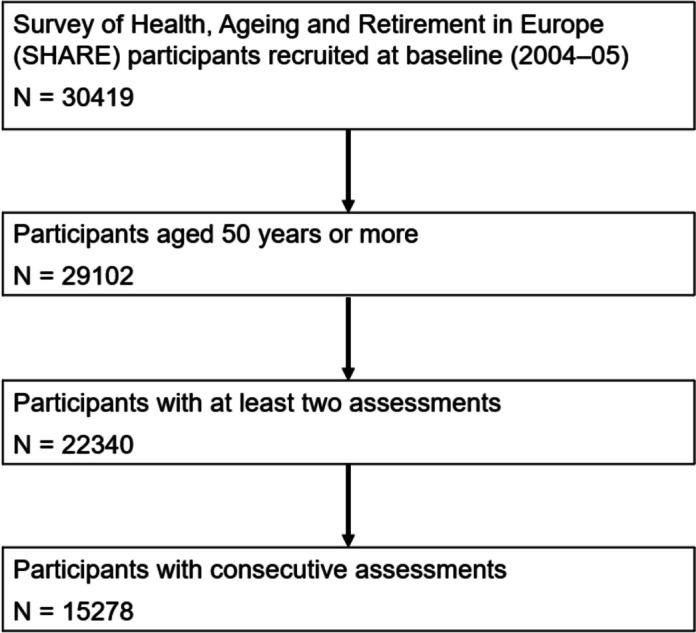
Flowchart for the Survey of Health, Ageing and Retirement in Europe (SHARE) participants

Of the initial sample of SHARE (*n* = 30,419), we restricted our analyses to participants aged 50 years and over at baseline (*n* = 29,102), who had consecutive follow-ups (*n* = 15,278) (Fig. 1). After running the clustering, the distribution between the clusters was uneven. At baseline, the first cluster (*n* = 11,369) and the second (*n* = 3374) encompassed the majority of the sample, leaving a small part for the third cluster (*n* = 535) (Table 1). Participants of the first and second clusters had similar baseline characteristics evoking healthy ageing. Participants of the third cluster were older (mean age 76.5 years [SD 11]), often female (*n* = 368 [68.6%]), had lower education level (*n* = 426 [79.6%] attained less than upper secondary education), more mobility impairment (mean mobility impairment score 4.9 [SD 1.5]), more functional impairment (mean Activities of Daily Living [ADL] score 3.1 [SD 1.7] and mean Instrumental Activities of Daily Living [IADL] score 4.2 [SD 1.8]), and more impaired cognition (mean immediate word recall test 2.6 [SD 1.9] and mean verbal fluency 10.4 [SD 6]) than participants of the first and second clusters at baseline. These characteristics corroborated that the third cluster was the one reflecting a high likelihood of dementia, thus named “Likely Dementia” cluster. Conversely, the first and second clusters’ participants were deemed dementia-free.

**Table 1:** Baseline characteristics of the SHARE study participants according to the three clusters identified by the algorithm

	SHARE		
	Cluster 1 (*n* = 11,369)	Cluster 2 (*n* = 3374)	Cluster 3 (*n* = 535)
Age, Years	64.6 (9.6)	65.2 (9.7)	76.5 (11)
Sex			
Female	6251 (55%)	1704 (50.5%)	368 (68.8%)
Male	5118 (45%)	1670 (49.5%)	167 (31.2%)
Education			
Less than upper secondary education	5632 (49.5%)	1834 (54.4%)	426 (79.6%)
Upper secondary and vocational training	3446 (30.3%)	928 (27.5%)	69 (12.9%)
Tertiary education	2291 (20.2%)	612 (18.1%)	40 (7.5%)
Mobilitity impairment score [0–7]	1 (1.4)	1 (1.4)	4.9 (1.5)
Autonomy			
ADL score [0–6]^a^	0.1 (0.3)	0.1 (0.4)	3.1 (1.7)
IADL score [0–7]^a^	0.2 (0.5)	0.2 (0.6)	4.2 (1.8)
Cognition			
Immediate Word Recall [0–10]^a^	5 (1.7)	4.7 (1.9)	2.6 (1.9)
Verbal Fluency [0–67]^a^	19.7 (7.1)	18.7 (7.3)	10.4 (6)

^a^ Missing values were imputed using MissMDA package

### Discrimination power

We compared our algorithm identification with the self-reported dementia diagnosis in the SHARE dataset, which was available from wave 2 (Table 2). Our clustering algorithm allowed the identification of a higher number of “Likely Dementia” cases compared with self-reported dementia cases. The AUC metric ranged from 0.754 (0.722–0.787) to 0.830 (0.800–0.861), suggesting good discrimination power. Sensitivity peaked at wave 4 reaching 0.714 (0.659–0.770) then slowly decreased after. Specificity remained high (> 0.9) in all waves. Results by country are given in Supplementary Information (Supplementary Table 3).

**Table 2:** Comparison of self-reported dementia cases and Cluster 3 “Likely Dementia” cases

Wave	Number of participants	SHARE								
		Clusters			Self-reported dementia			Metrics		
		Cluster 1	Cluster 2	Cluster 3 (Likely Dementia)	Missing	No	Yes	AUC (95% CI)	Sensitivity (95% CI)	Specificity (95% CI)
Wave 1 (2004–05)	15,278	11,369 (74.4%)	3374 (22.1%)	535 (3.5%)	NA	NA	NA	NA	NA	NA
Wave 2 (2006–07)	15,278	11,433 (74.8%)	2832 (18.5%)	1013 (6.6%)	40 (0.3%)	14,960 (97.9%)	278 (1.8%)	0.805 (0.776–0.835)	0.665 (0.610–0.721)	0.945 (0.942–0.949)
Wave 4 (2010–11)	10,008	7911 (79%)	1406 (14%)	691 (7%)	21 (0.2%)	9735 (97.3%)	252 (2.5%)	0.825 (0.794–0.855)	0.702 (0.646–0.759)	0.947 (0.943–0.952)
Wave 5 (2012–13)	8418	6429 (76.4%)	1312 (15.6%)	677 (8%)	10 (0.1%)	8129 (96.6%)	279 (3.3%)	0.794 (0.763–0.825)	0.649 (0.593–0.705)	0.939 (0.934–0.944)
Wave 6 (2014–15)	6485	4913 (75.8%)	987 (15.2%)	585 (9%)	8 (0.1%)	6204 (95.7%)	273 (4.2%)	0.755 (0.723–0.787)	0.579 (0.520–0.637)	0.931 (0.925–0.938)
Wave 7 (2016–17)	5533	3991 (72.1%)	1028 (18.6%)	514 (9.3%)	5 (0.1%)	5252 (94.9%)	276 (5%)	0.745 (0.712–0.777)	0.558 (0.499–0.617)	0.931 (0.925–0.938)

*AUC* Area Under the Curve, *CI* Confidence interval, *NA* Not available

### Effect of age and sex

**Fig. 2:**
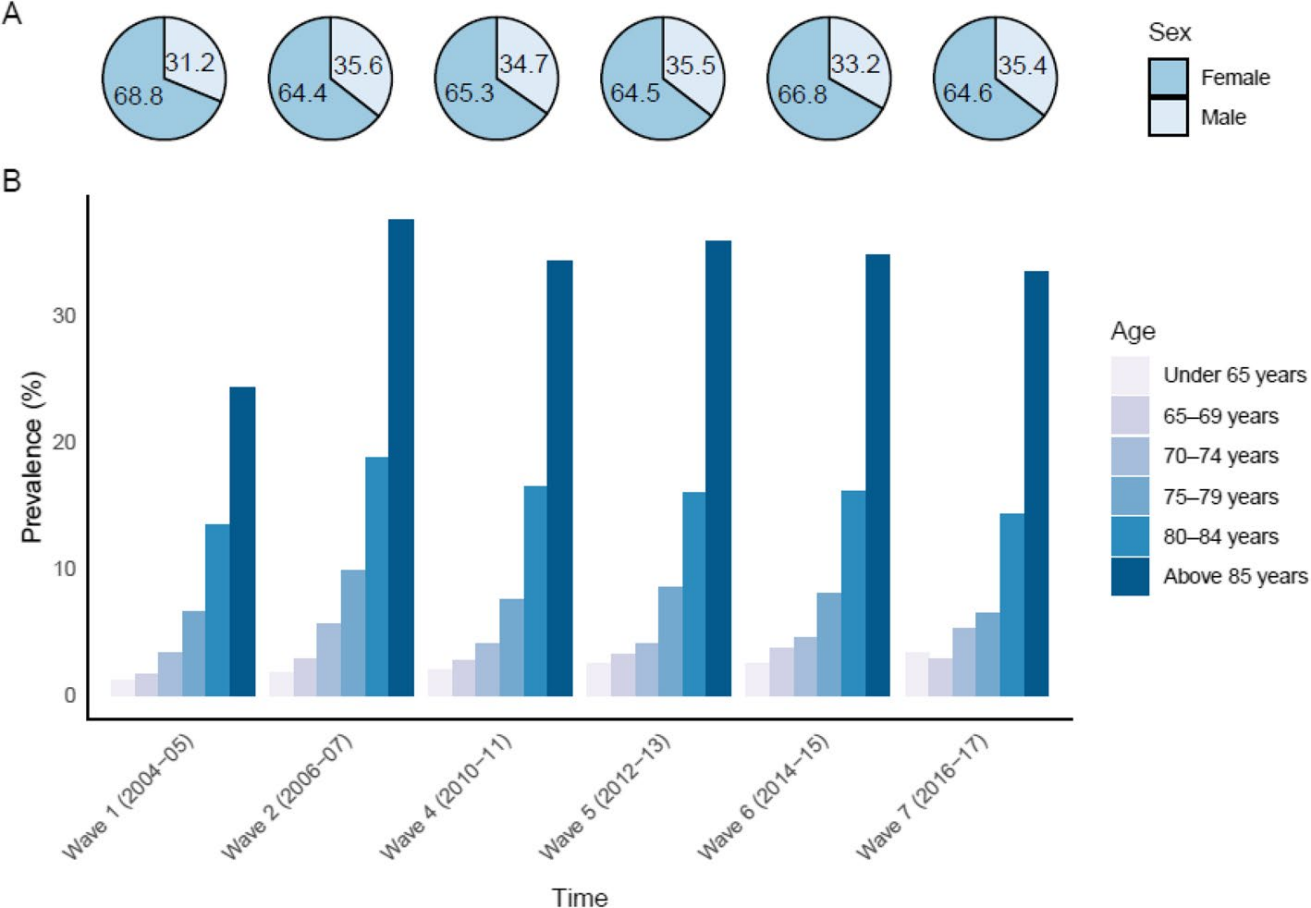
Prevalence of participants of the “Likely Dementia” cluster **A** by sex, and **B** by age

Older age and female sex were both associated with an increased risk of entering “Likely Dementia” cluster. The prevalence of “Likely Dementia” was higher in women with approximatively a 2:1 female to male ratio across all waves (Fig. 2A). The number of “Likely Dementia” cases increased with age (Fig. 2B). For instance, at wave 2, the prevalence of “Likely Dementia” cases gradually rose with age: 1.8% in those under 65 years, 3.1% in 65–69 years, 5.9% in 70–74 years, 10.2% in 75–79 years, 18.9% in 80–85 years, and 37.4% in more than 85 years old participants.

### Multistate models

**Fig. 3:**
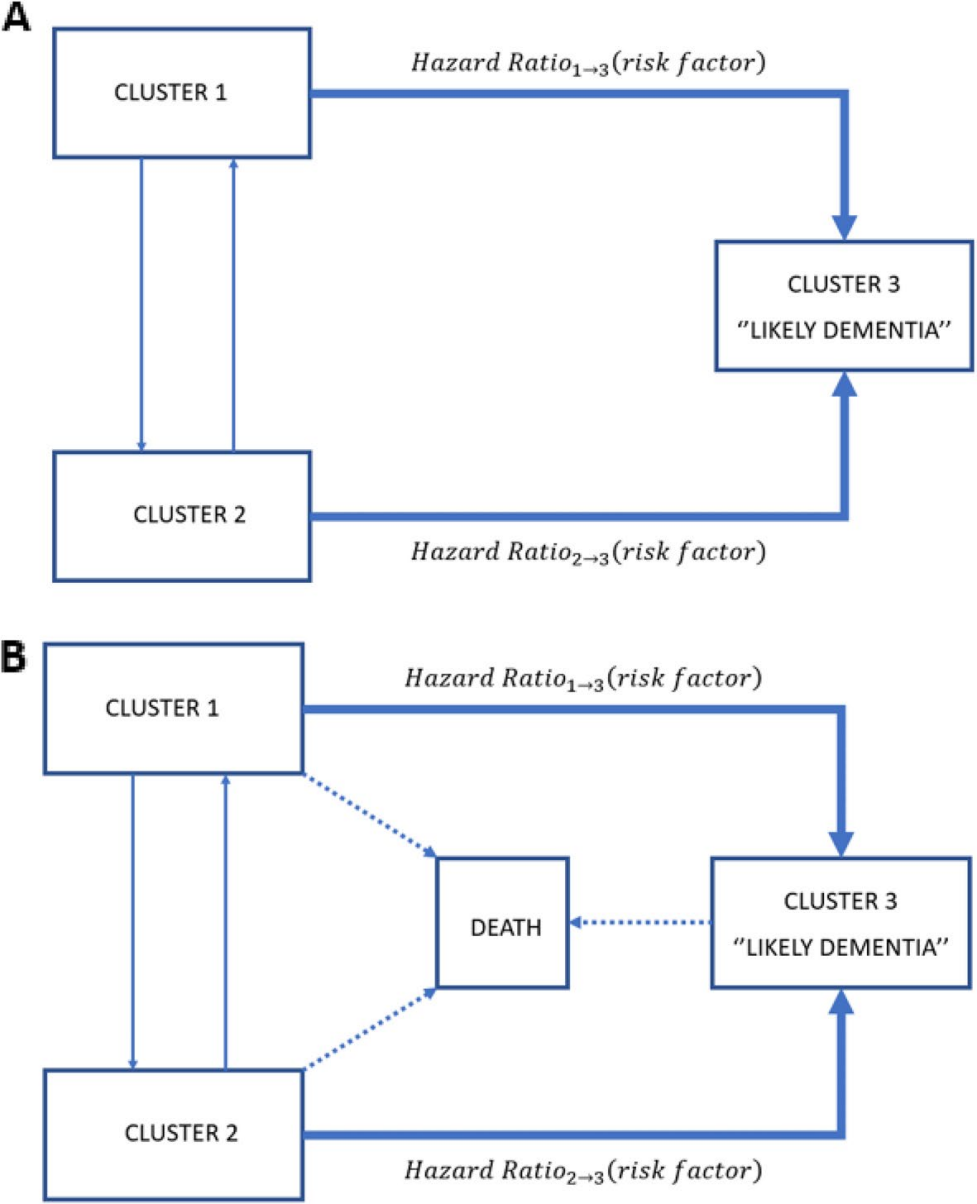
Three-state models **A** Multistate model **B** Multistate survival model

To assess the associations of dementia risk factors with the risk of transitioning to “Likely Dementia” cluster (Table 3), we computed a multistate model (Fig. 3A). Nine of the eleven dementia risk factors, chosen a priori, were associated with an increased risk of transition from Cluster 1 to “Likely Dementia” cluster: low education level (Hazard Ratio [HR] 1.92, 95%CI [1.58 − 2.33]), poor hearing (1.74 [1.45 − 2.09]), hypertension (1.35 [1.14 − 1.16]), smoking (1.45 [1.13 − 1.87]), depression (2.51 [1.06 − 3.07]), social isolation (1.66 [1.39 − 1.98]), physical inactivity (3.66 [2.97 − 4.51]), diabetes (2.4 [1.94 − 2.96]), and obesity (1.7 [1.39 − 2.07]). Some of these associations were also significant for transition from Cluster 2 to “Likely Dementia” cluster: depression (2.39 [1.62 − 3.53]), social isolation (2.31 [1.51 − 3.53]), physical inactivity (3.21 [2.12 − 4.87]), and obesity (1.58 [1.08 − 2.32]).

**Table 3:** Multistate models for the transition to cluster 3 (“Likely Dementia”)

	SHARE					
	Main analysis		Sensitivity analyses			
	Model 1 (*n* = 15,278)		Model 2 (*n* = 15,173)		Model 3 (*n* = 14,190)	
	HR (95% CI) (1 → 3)	HR (95% CI) (2 → 3)	HR (95% CI) (1 → 3)	HR (95% CI) (2 → 3)	HR (95% CI) (1 → 3)	HR (95% CI) (2 → 3)
Low education	1.92 (1.58–2.33)	1.32 (0.91–1.9)	1.86 (1.6–2.17)	1.18 (0.86–1.61)	1.77 (1.52–2.07)	0.97 (0.67–1.4)
Hearing loss	1.74 (1.45–2.09)	1.23 (0.85–1.79)	1.38 (1.2–1.59)	1.03 (0.75–1.42)	1.2 (1.03–1.4)	0.88 (0.59–1.32)
Hypertension	1.35 (1.14–1.16)	1.24 (0.9–1.72)	1.36 (1.2–1.55)	1.14 (0.86–1.5)	1.34 (1.17–1.53)	1.09 (0.76–1.55)
Drinking (> 21 units)	0.79 (0.55–1.14)	0.42 (0.16–1.09)	1.25 (0.99–1.58)	0.36 (0.13–1.04)	1.37 (1.09–1.73)	0.54 (0.21–1.41)
Smoking	1.45 (1.13–1.87)	1.29 (0.73–2.29)	1.64 (1.36–1.99)	2.23 (1.57–3.16)	1.7 (1.39–2.07)	2.68 (1.79–4.03)
Depression	2.51 (1.06–3.07)	2.39 (1.62–3.53)	2.05 (1.76–2.4)	1.98 (1.42–2.77)	1.78 (1.51–2.11)	1.65 (1.07–2.54)
Social isolation	1.66 (1.39–1.98)	2.31 (1.51–3.53)	1.61 (1.4–1.86)	1.6 (1.15–2.24)	1.56 (1.35–1.81)	1.15 (0.79–1.68)
Physical inactivity	3.66 (2.97–4.51)	3.21 (2.12–4.87)	2.48 (2.07–2.97)	2.89 (2–4.17)	2.09 (1.72–2.54)	1.33 (0.68–2.6)
Diabetes	2.4 (1.94–2.96)	1.32 (0.79–2.21)	2.15 (1.82–2.54)	1.32 (0.85–2.05)	2.22 (1.88–2.62)	0.88 (0.45–1.73)
Obesity	1.7 (1.39–2.07)	1.58 (1.08–2.32)	1.65 (1.41–1.93)	1.43 (1.01–2.01)	1.76 (1.5–2.06)	1.32 (0.83–2.1)
Air Pollution	0.84 (0.7–1.02)	1.26 (0.83–1.9)	0.92 (0.79–1.07)	1.32 (0.92–1.89)	0.94 (0.81–1.1)	1.32 (0.83–2.08)

Analyses using age as time-scale. All transitions were adjusted for sex. Transition towards the third cluster (“Likely Dementia”) was further adjusted for age and each risk factor individually. All risk factors were taken at baseline. Main analysis was based on a multistate model (Model 1). Sensitivity analyses were based on a multistate survival model with death as an absorbing state. First, 105 participants were removed because of inconsistencies of dates (Model 2). Second, cases identified either at the first or the second waves were removed (Model 3)

*HR* hazard ratio, *CI* Confidence interval

In the first sensitivity analysis which took into account death (Fig. 3B), we excluded 105 participants due to inconsistencies between interview and death dates. All of the above-described associations between dementia risk factors and transition to “Likely Dementia” cluster remained significant albeit with lower HR, except for hypertension. Of more, smoking became significantly associated with the risk of transition from Cluster 2 to “Likely Dementia” cluster (2.23 [1.57 − 3.16]). In the second sensitivity analysis, where prevalent and incident cases at wave 1 (2004 − 05) and wave 2 (2006 − 07) (*n* = 983) were further removed, HRs of transition from cluster 1 to “Likely Dementia” cluster did not change, but excessive alcohol drinking became a significant risk factor (1.34 [1.17 − 1.53]). As for transitions from Cluster 2 to “Likely Dementia” cluster, only smoking (2.68 [1.79 − 4.03]) and depression (1.65 [1.07 − 2.54]) remained significant.

### Replication in ELSA

Of the initial sample of ELSA (*n* = 12,099), we restricted our analyses to participants over 50 years at baseline (*n* = 11,522) and further restricted to participants who had consecutive follow-ups (*n* = 7840) (Supplementary Fig. 2). Overall, results obtained with ELSA participants were similar to those found in the SHARE cohort (Supplementary Table 4).

At baseline, participants of the third cluster (*n* = 659) were more likely older (mean age 69.8 [SD 11]), more likely female (*n* = 401 [60.8%]), of lower education level (*n* = 423 [64.2%] attained less than upper secondary education), had more mobility impairments (mean mobility impairment score 4.9 [SD 1.5]), more functional impairment (mean ADL score 2.7 [SD 1.5] and mean IADL score 2.5 [SD 1.4]), worse cognition (mean immediate word recall test 4.6 [SD 1.9], and mean verbal fluency 16.5 [SD 6]) than the other clusters.

Our clustering algorithm identified a higher number of “Likely Dementia” cases compared with self-reported dementia cases. Except for wave 1 (2002 − 03) in which the number of self-reported dementia cases was small (*n* = 24), the algorithm identification AUC metric values were similar to those found with SHARE (Supplementary Table 5). Sensitivity and specificity were balanced.

Women were more likely to be in the “Likely Dementia” group, and prevalence of “Likely Dementia” status rose with age (Supplementary Fig. 3).

Ten dementia risk factors were tested (not air pollution due to missing urbanicity data). Their associations with transition to “Likely Dementia” cluster remained similar to those found with the SHARE dataset (Supplementary Table 6) except for excessive alcohol drinking which was protective for the transition from Cluster 1 to “Likely Dementia” cluster (HR 0.6 [0.43 − 0.83]). Four risk factors were associated with an increased risk of transition from Cluster 2 to “Likely Dementia” cluster: hypertension (1.64 [1.13 − 2.38]), depression (2 [1.26 − 3.17]), physical inactivity (2.69 [1.73 − 4.18]), and diabetes (2.23 [1.26 − 3.95]). We did not take death into account in the multistate models as death data were not available for each wave in the sensitivity analysis.

Removing prevalent and incident cases at wave 1 (2002 − 03) and wave 2 (2004 − 05) in sensitivity analysis led to similar results with few exceptions. Excessive alcohol drinking was no longer significant for the transition from Cluster 1 to “Likely Dementia” cluster (0.79 [0.58 − 1.08]). Only physical inactivity remained significant for the risk of transition from Cluster 2 to “Likely Dementia” cluster (2.02 [1.1 − 3.69]).

## Discussion

Unsupervised clustering applied to two longitudinal population-based surveys of ageing (SHARE and ELSA) identified participants with high likelihood of dementia using longitudinal data related to functional and cognitive measurements. In both surveys, this method had a good discrimination performance when compared with self-reported diagnosis of dementia. “Likely Dementia” status was more common in older participants and in women with a 2:1 sex ratio. Low education, hearing loss, hypertension, smoking, depression, social isolation, physical inactivity, diabetes, and obesity were associated with a higher risk of subsequent transition to “Likely Dementia” cluster. Results for excessive alcohol drinking and air pollution were inconclusive. Applying clustering to longitudinal cohorts for the identification of high likelihood of dementia paves the way for researchers to conduct future secondary analyses on population ageing surveys worldwide.

Although supervised machine learning algorithms have already been used in population surveys to identify persons with dementia [17], they have their limitations, e.g., they require a subsample of data to be labelled “diagnosis of dementia”, and their external validity remains variable. Conversely, unsupervised machine learning may overcome such limitations as suggested in a previous cross-sectional study [7]. Here, we used an improved clustering method combining longitudinal data and a limited number of variables related to participants’ cognition and daily functions. Our clustering algorithm identified a greater number of people with a high likelihood of dementia in both SHARE and ELSA compared with self-reported dementia cases. Identifying a higher number of probable dementia cases in population ageing surveys might give a better statistical power to future studies of dementia determinants and outcomes. Moreover, this clustering method relies on cognitive and functional status data, largely available in HRS family studies and in several population ageing surveys, which makes it very suitable to apply to other ageing surveys including those in LMIC. Noteworthy, our study took into account many biases inherent to longitudinal studies, in particular attrition [18] due to loss to follow-up or death. Internal validity was assessed using different approaches: comparison with self-reported diagnosis of dementia, impact of age and sex on dementia prevalence, and impact of known dementia risk factor on the risk of being classified as a “Likely Dementia” case. Results were obtained using data of 12 countries participating in SHARE, and then replicated in ELSA.

On the other hand, one should carefully examine our results. For instance, detecting a “Likely Dementia” status by the algorithm cannot, by any stretch, be taken as a diagnosis of the disease without clinical validation. Future studies that compare our identification method with the recently developed cognitive assessment in HRS family cohorts using the Harmonized Cognitive Assessment Protocol (HCAP) [19] are warranted. Our method cannot distinguish the aetiology of dementia, whether Alzheimer’s disease (AD) or others. Contrary to the results of our prior cross-sectional study, Cluster 1 and Cluster 2 participants were similar in terms of daily function, cognition, and mobility, yet they differed in their risk of transition to Cluster 3 (“Likely Dementia”). However, we cannot rule out the possibility that the non-significant HRs observed for the transition from Cluster 2 to “Likely Dementia” cluster resulted from a lack of statistical power. Although this three-cluster partition remains consistent with our earlier work [7], future investigation will test the interest of further simplification by merging the first two clusters together. The lack of biological or imaging biomarkers in this study could also be seen as a limitation. Yet, biomarkers are often costly, expert-dependent, and rarely available in large population ageing studies. As for genetics, Apolipoprotein E (*APOE*) [20] and polygenic scores [21] are associated with a higher risk of AD, but the role of genetic factors in explaining future risk of dementia remains modest [21, 22]. The results for excessive alcohol drinking were ambiguous. We observed a deleterious drinking effect in SHARE, whereas it was protective in ELSA. Excessive drinking has been entangled for the brain damage it causes [23], yet its exact relationship with dementia risk is debated since alcohol thresholds and time of exposure differ between studies [24, 25]. The association between air pollution and dementia was inconclusive in SHARE and could not be explored in ELSA. Urbanicity (i.e., geographical variation between urban and rural areas) was used as a proxy for air pollution as proposed recently [12]. Yet, people living in rural areas have shown higher rates of dementia compared with their urban counterparts [26, 27]. Switching to quantifiable pollution markers (fine particulate matter or ozone) that have been linked to an increased risk of dementia [28] is more than desirable.

## Conclusion

Unsupervised clustering is an efficient method to detect people with probable dementia in population ageing surveys using their cognitive and functional characteristics in a longitudinal setting. This approach opens new perspectives for the analyses of population data sets already available worldwide in HIC and LMIC to better compare and understand dementia determinants and outcomes.

### Supplementary Information

**Additional file 1.**

## Data Availability

All the data we used are publicly available. We used the harmonised versions of SHARE and ELSA provided by the Gateway to Global Aging Data (https://g2aging.org/). ELSA raw data can be downloaded via the UK Data Service (https://ukdataservice.ac.uk/find-data/). SHARE raw data can be accessed on their website (http://www.share-project.org/data-access.html).

## Abbreviations

AD: Alzheimer’s disease
ADL: Activities of Daily Living
APOE: Apolipoprotein E
AUC: Area Under the Curve
BMI: Body Mass Index
CES-D: Centre for Epidemiologic Studies Depression
ELSA: English Longitudinal Study of Ageing
HCAP: Harmonized Cognitive Assessment Protocol
HCPC: Hierarchical Clustering on Principal Components
HIC: High income countries
HR: Hazard Ratio
HRS: Health and Retirement Study
IADL: Instrumental Activities of Daily Living
IPW: Inverse Probability Weighting
LMIC: Low and middle income countries
MELODEM: MEthods in LOngitudinal research on DEMentia
MFA: Multiple Factor Analysis
MND: Major neurocognitive disorder
SHARE: Survey of Health, Ageing, and Retirement in Europe
STROBE: STrengthening the Reporting of OBservational studies in Epidemiology
