# Supplementary material for: Identification of high likelihood of dementia in population-based surveys using unsupervised clustering: a longitudinal analysis
Source: Alzheimers Res Ther. 2023 Nov 29;15:209. doi: 10.1186/s13195-023-01357-9 (PMC10688099; doi:10.1186/s13195-023-01357-9)
Supplement: Supplementary file 1 — Additional file 1. [file 13195_2023_1357_MOESM1_ESM.docx]

**Alzheimer’s Research and Therapy – Supporting Information**

**Identification of high likelihood of dementia in population-based surveys using unsupervised clustering: a longitudinal analysis**

**Table of Contents**

[I. Appendix 2](#_Toc135066901)

[Multiple Factor Analysis Scientific Rationale 2](#_Toc135066902)

[II. Supplementary Figures 3](#_Toc135066903)

[S Fig 1. Data structure in SHARE 4](#_Toc135066904)

[S Fig 2. Flowchart for the English Longitudinal Study of Ageing (ELSA) participants 4](#_Toc135066905)

[S Fig 3. Prevalence of participants from the ‘’Likely dementia’’ cluster (A) by sex, and (B) by age in ELSA 5](#_Toc135066906)

[III. Supplementary Tables: 6](#_Toc135066907)

[S Table 1. Summary of variables used for both imputation and Multiple Factor analysis (MFA) in SHARE 7](#_Toc135066908)

[S Table 2. Summary of variables used for both imputation and Multiple Factor analysis (MFA) in ELSA 9](#_Toc135066909)

[S Table 3. Comparison of self-reported dementia cases and Cluster 3 "Likely Dementia" cases by country (SHARE) 12](#_Toc135066910)

[S Table 4. Baseline characteristics of the ELSA study participants according to the three clusters identified by the algorithm 13](#_Toc135066911)

[S Table 5. Comparison of self-reported dementia cases and Cluster 3 “Likely Dementia” cases in ELSA 14](#_Toc135066912)

[S Table 6. Multistate models for the transition to cluster 3 ("Likely Dementia") in ELSA 15](#_Toc135066913)

# Appendix

### Multiple Factor Analysis Scientific Rationale

Multiple Factor Analysis (MFA) is a principal component method similar to Principal Component Analysis (PCA) but, compared to PCA, it balances the influence of groups of variables. In our case, it balances the influence of two components, participants’ function and cognition, which have equal importance in the clinical definition of dementia (data structure for SHARE is given in S Fig 1). Then, as PCA, it gives a representation of individuals in such a way that individuals are close on the representation if they have close values from the point of view of all the variables of all the groups. Subsequently, clustering can be performed on the MFA results. Full description of the variables used for SHARE and ELSA studies are available in S Table 1 and 2, respectively.

# Supplementary Figures


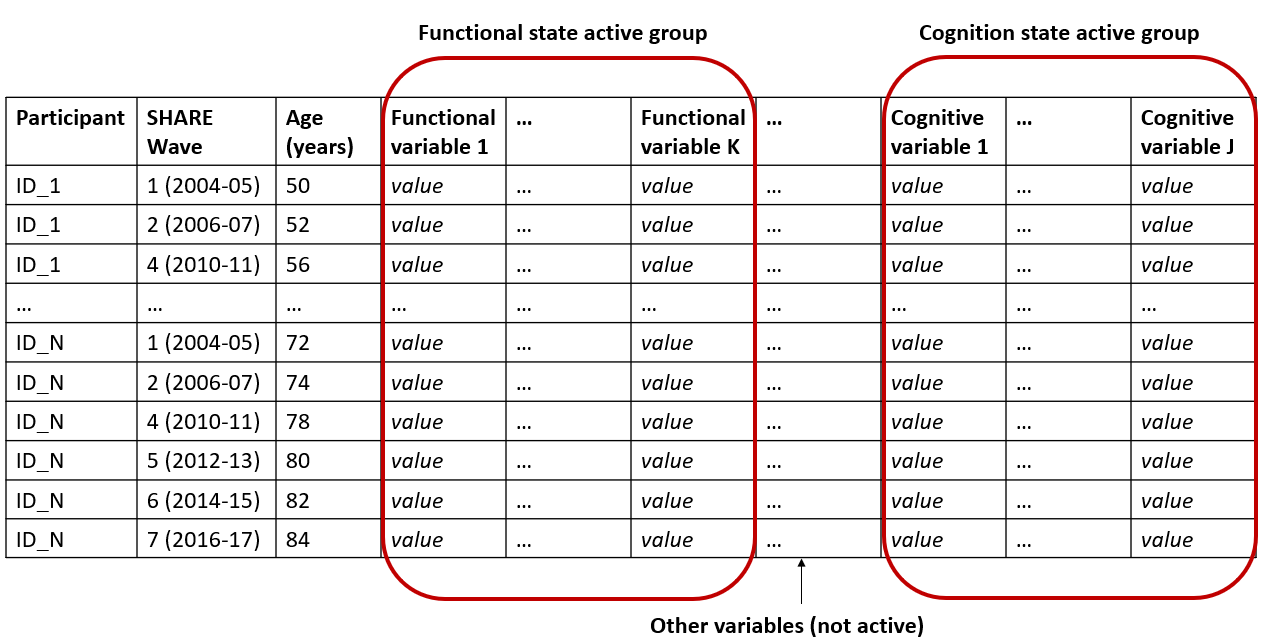


S Fig 1. Data structure in SHARE Each participant (from 1 to N) was seen multiple times (at least twice). Variables informative of functional status (from 1 to K) and variables informative of cognition (from 1 to J) formed the two active groups in the Multiple Factor Analysis (MFA).


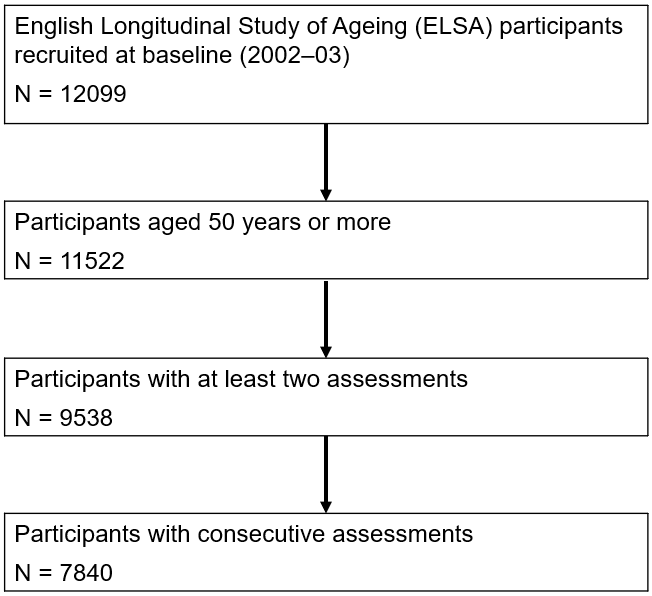


### S Fig 2. Flowchart for the English Longitudinal Study of Ageing (ELSA) participants


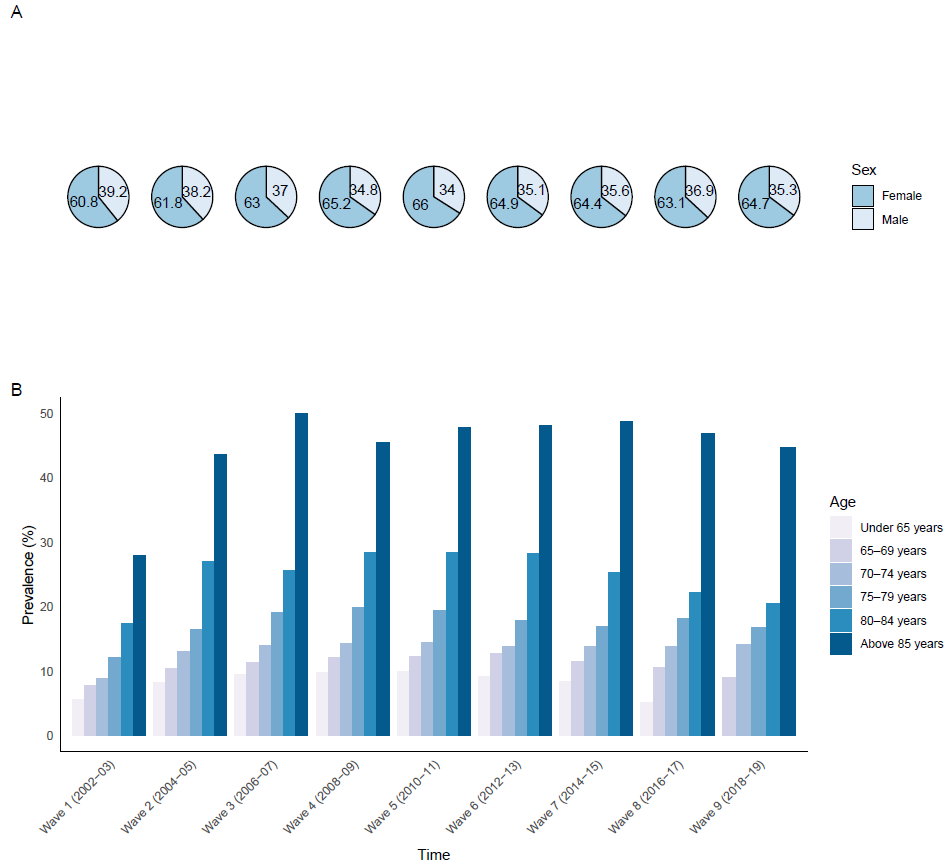


### S Fig 3. Prevalence of participants from the ‘’Likely dementia’’ cluster (A) by sex, and (B) by age in ELSA

# Supplementary Tables:

| **SHARE Database** | | | | | | | | | | |  |
| --- | --- | --- | --- | --- | --- | --- | --- | --- | --- | --- | --- |
| **Group** |  | **Variable** |  | **Description** |  | **Categories** |  | **Active groups** | | | |
|  |  |  |  |  |  |  |  | **Imputation** |  | **MFA** | |
|  |  |  |  |  |  |  |  |  |  |  | |
| Wave |  | wave |  | Wave of participation |  | 1. Wave 1 (2004–05) 2. Wave 2 (2006–07) 3. Wave 4 (2010–11) 4. Wave 5 (2012–13) 5. Wave 6 (2014–15) 6. Wave 7 (2016–17) |  | X |  |  | |
| Sex |  | ragender |  | Respondent gender |  | 1. Male 2. Female |  | X |  |  | |
| Age |  | age |  | (month/year of interview) - (month/year of birth) |  | . |  | X |  |  | |
| Education |  | raeducl |  | Respondent harmonized education level |  | 1. Less than upper secondary 2. Upper secondary and vocational training 3. Tertiary |  | X |  |  | |
| Autonomy (qualitative variables) |  | hlthlma |  | Respondent has health problem that limits activities |  | 0. Not limited 1. Limited |  | X |  | X | |
|  |  | walkra |  | Respondent has some difficulty walking across the room |  | 0. No 1. Yes |  |  |  |  |  |
|  |  | dressa |  | Respondent has some difficulty dressing |  | 0. No 1. Yes |  |  |  |  |  |
|  |  | batha |  | Respondent has some difficulty bathing/taking a shower |  | 0. No 1. Yes |  |  |  |  |  |
|  |  | eata |  | Respondent has some difficulty eating |  | 0. No 1. Yes |  |  |  |  |  |
|  |  | beda |  | Respondent has some difficulty getting in/out of the bed |  | 0. No 1. Yes |  |  |  |  |  |
|  |  | toilta |  | Respondent has some difficulty using the toilet |  | 0. No 1. Yes |  |  |  |  |  |
|  |  | phonea |  | Respondent has some difficulty using the telephone |  | 0. No 1. Yes |  |  |  |  |  |
|  |  | medsa |  | Respondent has some difficulty taking medications |  | 0. No 1. Yes |  |  |  |  |  |
|  |  | moneya |  | Respondent has some difficulty managing money |  | 0. No 1. Yes |  |  |  |  |  |
|  |  | shopa |  | Respondent has some difficulty buying grocery |  | 0. No 1. Yes |  |  |  |  |  |
|  |  | mealsa |  | Respondent has some difficulty preparing hot meal |  | 0. No 1. Yes |  |  |  |  |  |
|  |  | mapa |  | Respondent has some difficulty using a map |  | 0. No 1. Yes |  |  |  |  |  |
|  |  | housewka |  | Respondent has some difficulty doing household work around house |  | 0. No 1. Yes |  |  |  |  |  |
|  |  | walk100a |  | Respondent has some difficulty walking 100m |  | 0. No 1. Yes |  |  |  |  |  |
|  |  | sita |  | Respondent has some difficulty sitting for 2 hours |  | 0. No 1. Yes |  |  |  |  |  |
|  |  | chaira |  | Respondent has some difficulty getting up from chair |  | 0. No 1. Yes |  |  |  |  |  |
|  |  | climsa |  | Respondent has some difficulty climbing several flights of stairs |  | 0. No 1. Yes |  |  |  |  |  |
|  |  | clim1a |  | Respondent has some difficulty climbing one flight of stairs |  | 0. No 1. Yes |  |  |  |  |  |
|  |  | lifta |  | Respondent has some difficulty lifting/carrying 10lbs |  | 0. No 1. Yes |  |  |  |  |  |
|  |  | stoopa |  | Respondent has some difficulty stooping/kneeling/crouching |  | 0. No 1. Yes |  |  |  |  |  |
|  |  | armsa |  | Respondent has some difficulty reaching/extending arms up |  | 0. No 1. Yes |  |  |  |  |  |
|  |  | pusha |  | Respondent has some difficulty pushing/pulling long object |  | 0. No 1. Yes |  |  |  |  |  |
|  |  | dimea |  | Respondent has some difficulty picking up a small coin |  | 0. No 1. Yes |  |  |  |  |  |
| Autonomy (quantitative variables) |  | adltot_s |  | Some difficulty in Activities of Daily Living |  | . |  | X |  | X | |
|  |  | iadltot1_s |  | Any difficulty in total Instrumental Activities of Daily Living |  | . |  |  |  |  |  |
| Cognition (conditions) |  | cogimp |  | Whether factors impaired cognitive tests |  | 0. No 1. Yes |  | X |  | X | |
|  |  | cogothp |  | Whether other people present during cognitive tests |  | 0. No 1. Yes |  |  |  |  |  |
| Cognition (tests) |  | imrc |  | Immediate word recall |  |  |  | X |  | X | |
|  |  | dlrc |  | Delayed word recall |  | . |  |  |  |  |  |
|  |  | tr20 |  | Respondent recall summary score |  | . |  |  |  |  |  |
|  |  | verbf |  | Respondent verbal fluency score |  | . |  |  |  |  |  |
|  |  |  |  |  |  |  |  |  |  |  | |

### S Table 1. Summary of variables used for both imputation and Multiple Factor analysis (MFA) in SHARE

| **ELSA Database** | | | | | | | | | | |
| --- | --- | --- | --- | --- | --- | --- | --- | --- | --- | --- |
| Group |  | Variable |  | Description |  | Categories |  | Active groups | | |
|  |  |  |  |  |  |  |  | Imputation |  | MFA |
|  |  |  |  |  |  |  |  |  |  |  |
| Wave |  | wave |  | Wave of participation |  | 1. Wave 1 (2002–03) 2. Wave 2 (2004–05) 3. Wave 3 (2006–07) 4. Wave 4 (2008–09) 5. Wave 5 (2010–11) 6. Wave 6 (2012–13) 7. Wave 7 (2014–15) 8. Wave 8 (2016–17) 9. Wave 9 (2018–19) |  | X |  |  |
| Sex |  | ragender |  | Respondent gender |  | 1. Male 2. Female |  | X |  |  |
| Age |  | agey |  | Respondent age (years) at interview |  | . |  | X |  |  |
| Education |  | raeducl |  | Respondent harmonized education level |  | 1. Less than upper secondary 2. Upper secondary and vocational training 3. Tertiary |  | X |  |  |
| Autonomy (qualitative variables) |  | hlthlma |  | Respondent has health problem that limits activities |  | 0. Not limited 1. Limited |  | X |  | X |
|  |  | walkra |  | Respondent has some difficulty walking across the room |  | 0. No 1. Yes |  |  |  |  |
|  |  | dressa |  | Respondent has some difficulty dressing |  | 0. No 1. Yes |  |  |  |  |
|  |  | batha |  | Respondent has some difficulty bathing/taking a shower |  | 0. No 1. Yes |  |  |  |  |
|  |  | eata |  | Respondent has some difficulty eating |  | 0. No 1. Yes |  |  |  |  |
|  |  | beda |  | Respondent has some difficulty getting in/out of the bed |  | 0. No 1. Yes |  |  |  |  |
|  |  | toilta |  | Respondent has some difficulty using the toilet |  | 0. No 1. Yes |  |  |  |  |
|  |  | phonea |  | Respondent has some difficulty using the telephone |  | 0. No 1. Yes |  |  |  |  |
|  |  | medsa |  | Respondent has some difficulty taking medications |  | 0. No 1. Yes |  |  |  |  |
|  |  | moneya |  | Respondent has some difficulty managing money |  | 0. No 1. Yes |  |  |  |  |
|  |  | shopa |  | Respondent has some difficulty buying grocery |  | 0. No 1. Yes |  |  |  |  |
|  |  | mealsa |  | Respondent has some difficulty preparing hot meal |  | 0. No 1. Yes |  |  |  |  |
|  |  | mapa |  | Respondent has some difficulty using a map |  | 0. No 1. Yes |  |  |  |  |
|  |  | housewka |  | Respondent has some difficulty doing household work |  | 0. No 1. Yes |  |  |  |  |
|  |  | walk100a |  | Respondent has some difficulty walking 100m |  | 0. No 1. Yes |  |  |  |  |
|  |  | sita |  | Respondent has some difficulty sitting for 2 hours |  | 0. No 1. Yes |  |  |  |  |
|  |  | chaira |  | Respondent has some difficulty getting up from chair |  | 0. No 1. Yes |  |  |  |  |
|  |  | climsa |  | Respondent has some difficulty climbing several flights of stairs |  | 0. No 1. Yes |  |  |  |  |
|  |  | clim1a |  | Respondent has some difficulty climbing one flight of stairs (straight) |  | 0. No 1. Yes |  |  |  |  |
|  |  | lifta |  | Respondent has some difficulty lifting/carrying 10lbs |  | 0. No 1. Yes |  |  |  |  |
|  |  | stoopa |  | Respondent has some difficulty stooping/kneeling/crouching |  | 0. No 1. Yes |  |  |  |  |
|  |  | armsa |  | Respondent has some difficulty reaching/extending arms up |  | 0. No 1. Yes |  |  |  |  |
|  |  | pusha |  | Respondent has some difficulty pushing/pulling long object |  | 0. No 1. Yes |  |  |  |  |
|  |  | dimea |  | Respondent has some difficulty picking up a small coin |  | 0. No 1. Yes |  |  |  |  |
| Autonomy (quantitative variables) |  | adltot_s |  | Some difficulty in Activities of Daily Living |  | . |  | X |  | X |
|  |  | iadltot1_s |  | Any difficulty in total Instrumental Activities of Daily Living |  | . |  |  |  |  |
| Cognition (conditions) |  | cogimp |  | Whether factors impaired cognitive tests |  | 0. No 1. Yes |  | X |  | X |
|  |  | cogothp |  | Whether other people present during cognitive tests |  | 0. No 1. Yes |  |  |  |  |
| Cognition (tests) |  | imrc |  | Immediate word recall |  | . |  | X |  | X |
|  |  | dlrc |  | Delayed word recall |  | . |  |  |  |  |
|  |  | tr20 |  | Respondent recall summary score |  | . |  |  |  |  |
|  |  | verbf |  | Respondent verbal fluency score |  | . |  |  |  |  |
|  |  |  |  |  |  |  |  |  |  |  |

### S Table 2. Summary of variables used for both imputation and Multiple Factor analysis (MFA) in ELSA

|  |  |  |  |  |  |  |  |  |  |  |  |  |  | **SHARE** |  |  |  |  |  |  |  |  |
| --- | --- | --- | --- | --- | --- | --- | --- | --- | --- | --- | --- | --- | --- | --- | --- | --- | --- | --- | --- | --- | --- | --- |
| **Country** |  | **Wave** |  | **Number of participants** |  |  |  | **Clusters** |  |  |  | **Self-reported dementia** | | | | |  |  |  | **Metrics** |  |  |
|  |  |  |  |  |  | **Cluster 1** |  | **Cluster 2** |  | **Cluster 3**  **(Likely Dementia)** |  | **Missing** |  | **No** |  | **Yes** |  | **AUC**  **(95% CI)** |  | **Sensitivity**  **(95% CI)** |  | **Specificity**  **(95% CI)** |
|  |  |  |  |  |  |  |  |  |  |  |  |  |  |  |  |  |  |  |  |  |  |  |
| Austria |  | Wave 1  (2004–05) |  | 888 |  | 686  (77.3%) |  | 177  (19.9%) |  | 25  (2.8%) |  | NA |  | NA |  | NA |  | NA |  | NA |  | NA |
|  |  | Wave 2  (2006–07) |  | 888 |  | 704  (79.3%) |  | 132  (14.9%) |  | 52  (5.8%) |  | 1  (0.1%) |  | 870  (98%) |  | 17  (1.9%) |  | 0.707  (0.580–0.833) |  | 0.471  (0.233–0.708) |  | 0.943  (0.927–0.958) |
|  |  | Wave 4  (2010–11) |  | 549 |  | 436  (79.4%) |  | 84  (15.3%) |  | 29  (5.3%) |  | 4  (0.7%) |  | 525  (95.6%) |  | 20  (3.7%) |  | 0.746  (0.627–0.865) |  | 0.550  (0.332–0.768) |  | 0.943  (0.923–0.963) |
|  |  | Wave 5  (2012–13) |  | 455 |  | 365  (80.2%) |  | 52  (11.4%) |  | 38  (8.4%) |  | 4  (0.9%) |  | 423  (93%) |  | 28  (6.1%) |  | 0.729  (0.624–0.834) |  | 0.536  (0.351–0.720) |  | 0.922  (0.896–0.948) |
|  |  | Wave 6  (2014–15) |  | 382 |  | 306  (80.1%) |  | 40  (10.5%) |  | 36  (9.4%) |  | 0  (0%) |  | 348  (91.1%) |  | 34  (8.9%) |  | 0.757  (0.660–0.853) |  | 0.588  (0.423–0.754) |  | 0.925  (0.898–0.953) |
|  |  | Wave 7  (2016–17) |  | 312 |  | 248  (79.5%) |  | 34  (10.9%) |  | 30  (9.6%) |  | 0  (0%) |  | 287  (92%) |  | 25  (8%) |  | 0.700  (0.586–0.814) |  | 0.480  (0.284–0.676) |  | 0.920  (0.888–0.951) |
|  |  |  |  |  |  |  |  |  |  |  |  |  |  |  |  |  |  |  |  |  |  |  |
| Belgium |  | Wave 1  (2004–05) |  | 2,471 |  | 1740  (70.4%) |  | 653  (26.4%) |  | 78  (3.2%) |  | NA |  | NA |  | NA |  | NA |  | NA |  | NA |
|  |  | Wave 2  (2006–07) |  | 2,471 |  | 1793  (72.6%) |  | 567  (22.9%) |  | 111  (4.5%) |  | 1  (0%) |  | 2442  (98.8%) |  | 28  (1.2%) |  | 0.798  (0.705–0.891) |  | 0.643  (0.465–0.820) |  | 0.953  (0.945–0.961) |
|  |  | Wave 4  (2010–11) |  | 1,779 |  | 1380  (77.6%) |  | 306  (17.2%) |  | 93  (5.2%) |  | 0  (0%) |  | 1748  (98.3%) |  | 31  (1.7%) |  | 0.781  (0.690–0.872) |  | 0.613  (0.441–0.784) |  | 0.949  (0.939–0.959) |
|  |  | Wave 5  (2012–13) |  | 1,546 |  | 1149  (74.3%) |  | 316  (20.5%) |  | 81  (5.2%) |  | 2  (0.1%) |  | 1502  (97.2%) |  | 42  (2.7%) |  | 0.710  (0.628–0.791) |  | 0.476  (0.325–0.627) |  | 0.943  (0.932–0.955) |
|  |  | Wave 6  (2014–15) |  | 1,357 |  | 1026  (75.6%) |  | 240  (17.7%) |  | 91  (6.7%) |  | 1  (0.1%) |  | 1305  (96.2%) |  | 51  (3.7%) |  | 0.690  (0.615–0.765) |  | 0.451  (0.314–0.588) |  | 0.929  (0.915–0.943) |
|  |  | Wave 7  (2016–17) |  | 1,169 |  | 866  (74.1%) |  | 231  (19.8%) |  | 72  (6.1%) |  | 1  (0.1%) |  | 1116  (95.5%) |  | 52  (4.4%) |  | 0.688  (0.614–0.763) |  | 0.442  (0.307–0.577) |  | 0.935  (0.920–0.949) |
|  |  |  |  |  |  |  |  |  |  |  |  |  |  |  |  |  |  |  |  |  |  |  |
| Denmark |  | Wave 1  (2004–05) |  | 1,073 |  | 830  (77.4%) |  | 216  (20.1%) |  | 27  (2.5%) |  | NA |  | NA |  | NA |  | NA |  | NA |  | NA |
|  |  | Wave 2  (2006–07) |  | 1,073 |  | 889  (82.9%) |  | 125  (11.6%) |  | 59  (5.5%) |  | 0  (0%) |  | 1,058 (98.6%) |  | 15  (1.4%) |  | 0.876  (0.769–0.984) |  | 0.811  (0.620–1.000)* |  | 0.953  (0.940–0.966) |
|  |  | Wave 4  (2010–11) |  | 776 |  | 661  (85.2%) |  | 84  (10.8%) |  | 31  (4%) |  | 2  (0.3%) |  | 762  (98.2%) |  | 12  (1.5%) |  | 0.814  (0.674–0.954) |  | 0.667  (0.400–0.933) |  | 0.962  (0.948–0.976) |
|  |  | Wave 5  (2012–13) |  | 682 |  | 574  (84.2%) |  | 75  (11%) |  | 33  (4.8%) |  | 0  (0%) |  | 669  (98.1%) |  | 13  (1.9%) |  | 0.822  (0.689–0.956) |  | 0.692  (0.441–0.943) |  | 0.952  (0.936–0.968) |
|  |  | Wave 6  (2014–15) |  | 603 |  | 505  (83.8%) |  | 73  (12.1%) |  | 25  (4.1%) |  | 2  (0.3%) |  | 586  (97.2%) |  | 15  (2.5%) |  | 0.711  (0.577–0.846) |  | 0.467  (0.214–0.719) |  | 0.956  (0.939–0.972) |
|  |  | Wave 7  (2016–17) |  | 514 |  | 384  (74.7%) |  | 101  (19.7%) |  | 29  (5.6%) |  | 0  (0.0%) |  | 501  (97.5%) |  | 13  (2.5%) |  | 0.783  (0.641–0.925) |  | 0.615  (0.351–0.880) |  | 0.950  (0.931–0.969) |
|  |  |  |  |  |  |  |  |  |  |  |  |  |  |  |  |  |  |  |  |  |  |  |
| France |  | Wave 1  (2004–05) |  | 1661 |  | 1182  (71.2%) |  | 424  (25.5%) |  | 55  (3.3%) |  | NA |  | NA |  | NA |  | NA |  | NA |  | NA |
|  |  | Wave 2  (2006–07) |  | 1661 |  | 1246  (75%) |  | 334  (20.1%) |  | 81  (4.9%) |  | 22  (1.3%) |  | 1607  (96.8%) |  | 32  (1.9%) |  | 0.822  (0.736–0.907) |  | 0.688  (0.527–0.848) |  | 0.956  (0.946–0.966) |
|  |  | Wave 4  (2010–11) |  | 1153 |  | 935  (81.1%) |  | 141  (12.2%) |  | 77  (6.7%) |  | 10  (0.9%) |  | 1108  (96.1%) |  | 35  (3%) |  | 0.814  (0.730–0.898) |  | 0.686  (0.532–0.840) |  | 0.942  (0.929–0.956) |
|  |  | Wave 5  (2012–13) |  | 899 |  | 737  (82%) |  | 102  (11.3%) |  | 60  (6.7%) |  | 2  (0.2%) |  | 865  (96.2%) |  | 32  (3.6%) |  | 0.877  (0.801–0.952) |  | 0.812  (0.677–0.948) |  | 0.941  (0.925–0.957) |
|  |  | Wave 6  (2014–15) |  | 709 |  | 543  (76.6%) |  | 132  (18.6%) |  | 34  (4.8%) |  | 1  (0.1%) |  | 692  (97.6%) |  | 16  (2.3%) |  | 0.812  (0.689–0.935) |  | 0.688  (0.460–0.915) |  | 0.936  (0.918–0.955) |
|  |  | Wave 7  (2016–17) |  | 578 |  | 425  (73.5%) |  | 128  (22.2%) |  | 25  (4.3%) |  | 1  (0.2%) |  | 560  (96.9%) |  | 17  (2.9%) |  | 0.832  (0.715–0.948) |  | 0.706  (0.489–0.922) |  | 0.957  (0.940–0.974) |
|  |  |  |  |  |  |  |  |  |  |  |  |  |  |  |  |  |  |  |  |  |  |  |
| Germany |  | Wave 1  (2004–05) |  | 1,509 |  | 1169  (77.5%) |  | 300  (19.9%) |  | 40  (2.6%) |  | NA |  | NA |  | NA |  | NA |  | NA |  | NA |
|  |  | Wave 2  (2006–07) |  | 1,509 |  | 1136  (75.3%) |  | 303  (20.1%) |  | 70  (4.6%) |  | 2  (0.1%) |  | 1473  (97.6%) |  | 34  (2.3%) |  | 0.802  (0.716–0.887) |  | 0.647  (0.486–0.808) |  | 0.957  (0.946–0.967) |
|  |  | Wave 4  (2010–11) |  | 903 |  | 725  (80.3%) |  | 130  (14.4%) |  | 48  (5.3%) |  | 0  (0.0%) |  | 884  (97.9%) |  | 19  (2.1%) |  | 0.845  (0.739–0.951) |  | 0.737  (0.539–0.935) |  | 0.952  (0.938–0.967) |
|  |  | Wave 5  (2012–13) |  | 621 |  | 467  (75.2%) |  | 118  (19%) |  | 36  (5.8%) |  | 0  (0.0%) |  | 605  (97.4%) |  | 16  (2.6%) |  | 0.783  (0.655–0.911) |  | 0.625  (0.388–0.862) |  | 0.940  (0.922–0.959) |
|  |  | Wave 6  (2014–15) |  | 562 |  | 425  (75.6%) |  | 106  (18.9%) |  | 31  (5.5%) |  | 0  (0.0%) |  | 539  (95.9%) |  | 23  (4.1%) |  | 0.732  (0.620–0.844) |  | 0.522  (0.318–0.726) |  | 0.942  (0.923–0.962) |
|  |  | Wave 7  (2016–17) |  | 482 |  | 376  (78%) |  | 81  (16.8%) |  | 25  (5.2%) |  | 0  (0.0%) |  | 459  (95.2%) |  | 23  (4.8%) |  | 0.753  (0.641–0.865) |  | 0.565  (0.363–0.768) |  | 0.941  (0.920–0.963) |
|  |  |  |  |  |  |  |  |  |  |  |  |  |  |  |  |  |  |  |  |  |  |  |
| Greece |  | Wave 1  (2004–05) |  | 742 |  | 593  (79.9%) |  | 83  (11.2%) |  | 66  (8.9%) |  | NA |  | NA |  | NA |  | NA |  | NA |  | NA |
|  |  | Wave 2  (2006–07) |  | 742 |  | 621  (83.7%) |  | 46  (6.2%) |  | 75  (10.1%) |  | 3  (0.4%) |  | 724  (97.6%) |  | 15  (2%) |  | 0.747  (0.612–0.882) |  | 0.600  (0.352–0.848) |  | 0.894  (0.871–0.916) |
|  |  |  |  |  |  |  |  |  |  |  |  |  |  |  |  |  |  |  |  |  |  |  |
| Israel |  | Wave 1  (2004–05) |  | 241 |  | 140  (58.1%) |  | 74  (30.7%) |  | 27  (11.2%) |  | NA |  | NA |  | NA |  | NA |  | NA |  | NA |
|  |  | Wave 2  (2006–07) |  | 241 |  | 125  (51.9%) |  | 50  (20.7%) |  | 66  (27.4%) |  | 5  (2.1%) |  | 214  (88.8%) |  | 22  (9.1%) |  | 0.806  (0.705–0.906) |  | 0.873  (0.713–1.000)* |  | 0.748  (0.689–0.806) |
|  |  |  |  |  |  |  |  |  |  |  |  |  |  |  |  |  |  |  |  |  |  |  |
| Italy |  | Wave 1  (2004–05) |  | 1471 |  | 1048  (71.2%) |  | 351  (23.9%) |  | 72  (4.9%) |  | NA |  | NA |  | NA |  | NA |  | NA |  | NA |
|  |  | Wave 2  (2006–07) |  | 1471 |  | 1090  (74.1%) |  | 273  (18.6%) |  | 108  (7.3%) |  | 0  (0%) |  | 1449  (98.5%) |  | 22  (1.5%) |  | 0.755  (0.646–0.865) |  | 0.591  (0.385–0.796) |  | 0.920  (0.906–0.934) |
|  |  | Wave 4  (2010–11) |  | 1165 |  | 918  (78.8%) |  | 153  (13.1%) |  | 94  (8.1%) |  | 1  (0.1%) |  | 1134  (97.3%) |  | 30  (2.6%) |  | 0.879  (0.804–0.953) |  | 0.833  (0.700–0.967) |  | 0.924  (0.909–0.940) |
|  |  | Wave 5  (2012–13) |  | 1029 |  | 774  (75.2%) |  | 171  (16.6%) |  | 84  (8.2%) |  | 0  (0%) |  | 988  (96%) |  | 41  (4%) |  | 0.825  (0.749–0.902) |  | 0.732  (0.596–0.867) |  | 0.919  (0.902–0.936) |
|  |  | Wave 6  (2014–15) |  | 924 |  | 709  (76.7%) |  | 145  (15.7%) |  | 70  (7.6%) |  | 1  (0.1%) |  | 879  (95.1%) |  | 44  (4.8%) |  | 0.756  (0.674–0.837) |  | 0.591  (0.446–0.736) |  | 0.920  (0.902–0.938) |
|  |  | Wave 7  (2016–17) |  | 819 |  | 586  (71.6%) |  | 159  (19.4%) |  | 74  (9%) |  | 0  (0%) |  | 765  (93.4%) |  | 54  (6.6%) |  | 0.774  (0.700–0.848) |  | 0.630  (0.501–0.758) |  | 0.919  (0.900–0.938) |
|  |  |  |  |  |  |  |  |  |  |  |  |  |  |  |  |  |  |  |  |  |  |  |
| The Netherlands |  | Wave 1  (2004–05) |  | 1678 |  | 1212  (72.2%) |  | 439  (26.2%) |  | 27  (1.6%) |  | NA |  | NA |  | NA |  | NA |  | NA |  | NA |
|  |  | Wave 2  (2006–07) |  | 1678 |  | 1206  (71.9%) |  | 432  (25.7%) |  | 40  (2.4%) |  | 3  (0.2%) |  | 1655  (98.6%) |  | 20  (1.2%) |  | 0.687  (0.576–0.798) |  | 0.400  (0.185–0.615) |  | 0.974  (0.966–0.982) |
|  |  | Wave 4  (2010–11) |  | 1124 |  | 905  (80.5%) |  | 184  (16.4%) |  | 35  (3.1%) |  | 2  (0.2%) |  | 1101  (97.9%) |  | 21  (1.9%) |  | 0.700  (0.589–0.811) |  | 0.429  (0.217–0.640) |  | 0.972  (0.962–0.982) |
|  |  | Wave 5  (2012–13) |  | 951 |  | 721  (75.8%) |  | 201  (21.1%) |  | 29  (3.1%) |  | 1  (0.1%) |  | 926  (97.4%) |  | 24  (2.5%) |  | 0.780  (0.676–0.883) |  | 0.583  (0.386–0.781) |  | 0.976  (0.966–0.986) |
|  |  |  |  |  |  |  |  |  |  |  |  |  |  |  |  |  |  |  |  |  |  |  |
| Spain |  | Wave 1  (2004–05) |  | 1239 |  | 878  (70.9%) |  | 283  (22.8%) |  | 78  (6.3%) |  | NA |  | NA |  | NA |  | NA |  | NA |  | NA |
|  |  | Wave 2  (2006–07) |  | 1239 |  | 832  (67.1%) |  | 287  (23.2%) |  | 120  (9.7%) |  | 1  (0.1%) |  | 1204  (97.2%) |  | 34  (2.7%) |  | 0.860  (0.788–0.933) |  | 0.824  (0.695–0.952) |  | 0.897  (0.880–0.914) |
|  |  | Wave 4  (2010–11) |  | 942 |  | 601  (63.8%) |  | 219  (23.2%) |  | 122  (13%) |  | 1  (0.1%) |  | 892  (94.7%) |  | 49  (5.2%) |  | 0.893  (0.840–0.946) |  | 0.898  (0.813–0.983) |  | 0.888  (0.867–0.909) |
|  |  | Wave 5  (2012–13) |  | 848 |  | 506  (59.7%) |  | 218  (25.7%) |  | 124  (14.6%) |  | 0  (0%) |  | 795  (93.8%) |  | 53  (6.2%) |  | 0.825  (0.758–0.891) |  | 0.792  (0.683–0.902) |  | 0.857  (0.832–0.881) |
|  |  | Wave 6  (2014–15) |  | 725 |  | 470  (64.8%) |  | 150  (20.7%) |  | 105  (14.5%) |  | 0  (0%) |  | 669  (92.3%) |  | 56  (7.7%) |  | 0.811  (0.742–0.879) |  | 0.768  (0.657–0.878) |  | 0.854  (0.827–0.880) |
|  |  | Wave 7  (2016–17) |  | 619 |  | 382  (61.7%) |  | 152  (24.6%) |  | 85  (13.7%) |  | 2  (0.3%) |  | 562  (90.8%) |  | 55  (8.9%) |  | 0.743  (0.664–0.821) |  | 0.655  (0.529–0.780) |  | 0.831  (0.800–0.862) |
|  |  |  |  |  |  |  |  |  |  |  |  |  |  |  |  |  |  |  |  |  |  |  |
| Sweden |  | Wave 1  (2004–05) |  | 1652 |  | 1345  (81.4%) |  | 272  (16.5%) |  | 35  (2.1%) |  | NA |  | NA |  | NA |  | NA |  | NA |  | NA |
|  |  | Wave 2  (2006–07) |  | 1652 |  | 1337  (80.9%) |  | 252  (15.3%) |  | 63  (3.8%) |  | 2  (0.1%) |  | 1618  (98%) |  | 32  (1.9%) |  | 0.831  (0.747–0.915) |  | 0.688  (0.527–0.848) |  | 0.974  (0.966–0.982) |
|  |  | Wave 4  (2010–11) |  | 1131 |  | 978  (86.5%) |  | 104  (9.2%) |  | 49  (4.3%) |  | 1  (0.1%) |  | 1106  (97.8%) |  | 24  (2.1%) |  | 0.838  (0.742–0.934) |  | 0.708  (0.526–0.890) |  | 0.967  (0.957–0.978) |
|  |  | Wave 5  (2012–13) |  | 964 |  | 866  (89.8%) |  | 64  (6.7%) |  | 34  (3.5%) |  | 1  (0.1%) |  | 939  (97.4%) |  | 24  (2.5%) |  | 0.774  (0.669–0.878) |  | 0.583  (0.386–0.781) |  | 0.964  (0.952–0.976) |
|  |  | Wave 6  (2014–15) |  | 847 |  | 711  (84%) |  | 107  (12.6%) |  | 29  (3.4%) |  | 3  (0.3%) |  | 817  (96.5%) |  | 27  (3.2%) |  | 0.741  (0.640–0.842) |  | 0.519  (0.330–0.707) |  | 0.963  (0.950–0.976) |
|  |  | Wave 7  (2016–17) |  | 713 |  | 555  (77.9%) |  | 135  (18.9%) |  | 23  (3.2%) |  | 1  (0.1%) |  | 685  (96.1%) |  | 27  (3.8%) |  | 0.705  (0.604–0.805) |  | 0.444  (0.257–0.632) |  | 0.965  (0.951–0.979) |
|  |  |  |  |  |  |  |  |  |  |  |  |  |  |  |  |  |  |  |  |  |  |  |
| Switzerland |  | Wave 1  (2004–05) |  | 653 |  | 546  (83.6%) |  | 102  (15.6%) |  | 5  (0.8%) |  | NA |  | NA |  | NA |  | NA |  | NA |  | NA |
|  |  | Wave 2  (2006–07) |  | 653 |  | 560  (85.8%) |  | 80  (12.3%) |  | 13  (2%) |  | 0  (0%) |  | 646  (98.9%) |  | 7  (1.1%) |  | 0.777  (0.589–0.965) |  | 0.571  (0.205–0.938) |  | 0.983  (0.973–0.993) |
|  |  | Wave 4  (2010–11) |  | 486 |  | 450  (92.6%) |  | 26  (5.3%) |  | 10  (2.1%) |  | 0  (0%) |  | 475  (97.7%) |  | 11  (2.3%) |  | 0.766  (0.614–0.919) |  | 0.545  (0.251–0.840) |  | 0.987  (0.977–0.997) |
|  |  | Wave 5  (2014–15) |  | 376 |  | 330  (87.8%) |  | 38  (10.1%) |  | 8  (2.1%) |  | 0  (0%) |  | 369  (98.1%) |  | 7  (1.9%) |  | 0.632  (0.457–0.807) |  | 0.341  (0.075–0.611)* |  | 0.978  (0.963–0.993) |
|  |  | Wave 6  (2016–17) |  | 327 |  | 281  (85.9%) |  | 39  (11.9%) |  | 7  (2.1%) |  | 0  (0%) |  | 317  (96.9%) |  | 10  (3.1%) |  | 0.689  (0.529–0.849) |  | 0.400  (0.096–0.704) |  | 0.978  (0.962–0.994) |
|  |  | Wave 7  (2016–17) |  | 423 |  | 389  (92%) |  | 28  (6.6%) |  | 6  (1.4%) |  | 0  (0%) |  | 417  (98.6%) |  | 6  (1.4%) |  | 0.575  (0.420–0.730) |  | 0.237  (0.076–0.544)* |  | 0.983  (0.971–0.996) |

S Table 3. Comparison of self-reported dementia cases and Cluster 3 "Likely Dementia" cases by country (SHARE) *Values obtained using bootstrapping. *AUC* Area Under the Curve, *CI* confidence interval, *NA* not available

|  |  |  | **ELSA** |  |  |
| --- | --- | --- | --- | --- | --- |
|  | **Cluster 1** (n=6556) |  | **Cluster 2** (n=625) |  | **Cluster 3** (n=659) |
|  |  |  |  |  |  |
| Age, Years | 64.1 (9.7) |  | 65.8 (10.4) |  | 69.8 (11) |
| Sex |  |  |  |  |  |
| Female | 3584 (54.6%) |  | 322 (51.5%) |  | 401 (60.8%) |
| Male | 2976 (45.4%) |  | 303 (48.5%) |  | 258 (39.2%) |
| Education |  |  |  |  |  |
| Less than upper secondary education | 2646 (40.4%) |  | 345 (55.2%) |  | 423 (64.2%) |
| Upper secondary and vocational training | 3010 (45.9%) |  | 247 (39.5%) |  | 208 (31.6%) |
| Tertiary education | 900 (13.7%) |  | 33 (5.3%) |  | 28 (4.2%) |
| Mobility difficulty score [0–7] | 1.1 (1.4) |  | 1.6 (1.8) |  | 4.9 (1.4) |
| Autonomy |  |  |  |  |  |
| ADL score [0–6] | 0.1 (0.4) |  | 0.3 (0.7) |  | 2.7 (1.5) |
| IADL score [0–7] | 0.1 (0.4) |  | 0.3 (0.7) |  | 2.5 (1.4) |
| Cognition |  |  |  |  |  |
| Immediate Word Recall [0–10] | 5.7 (1.7) |  | 5.2 (1.8) |  | 4.6 (1.9) |
| Verbal Fluency [0–49] | 20.2 (6.1) |  | 18.7 (6.4) |  | 16.5 (6) |
|  |  |  |  |  |  |
| S Table 4. Baseline characteristics of the ELSA study participants according to the three clusters identified by the algorithm | | | | | |

|  |  |  |  |  |  |  |  |  |  |  | **ELSA** |  |  |  |  |  |  |  |  |
| --- | --- | --- | --- | --- | --- | --- | --- | --- | --- | --- | --- | --- | --- | --- | --- | --- | --- | --- | --- |
|  | **Number of participants** |  |  |  | **Clusters** |  |  |  | **Self-reported dementia** | | | | |  |  |  | **Metrics** |  |  |
| **Wave** |  |  | **Cluster 1** |  | **Cluster 2** |  | **Cluster 3**  **(Likely Dementia)** |  | **Missing** |  | **No** |  | **Yes** |  | **AUC**  **(95% CI)** |  | **Sensitivity**  **(95% CI)** |  | **Specificity**  **(95% CI)** |
|  |  |  |  |  |  |  |  |  |  |  |  |  |  |  |  |  |  |  |  |
| Wave 1  (2002–03) | 7840 |  | 6556  (83.6%) |  | 625  (8%) |  | 659  (8.4%) |  | 3  (0%) |  | 7813  (99.7%) |  | 24 (0.3%) |  | 0.646  (0.546–0.746) |  | 0.375  (0.181–0.569) |  | 0.917  (0.911–0.923) |
| Wave 2  (2004–05) | 7840 |  | 6210  (79.2%) |  | 566  (7.2%) |  | 1064  (13.6%) |  | 0  (0%) |  | 7778  (99.2%) |  | 62 (0.8%) |  | 0.749  (0.685–0.813) |  | 0.629  (0.509–0.749) |  | 0.868  (0.861–0.876) |
| Wave 3  (2006–07) | 6662 |  | 5204  (78.6%) |  | 380  (5.7%) |  | 1038  (15.7%) |  | 0  (0%) |  | 6550  (98.9%) |  | 72 (1.1%) |  | 0.814  (0.762–0.866) |  | 0.778  (0.682–0.874) |  | 0.850  (0.841–0.859) |
| Wave 4  (2008–09) | 5720 |  | 4468  (78.1%) |  | 292  (5.1%) |  | 960  (16.8%) |  | 0  (0%) |  | 5630  (98.4%) |  | 90 (1.6%) |  | 0.832  (0.788–0.877) |  | 0.822  (0.743–0.901) |  | 0.843  (0.833–0.852) |
| Wave 5  (2010–11) | 5125 |  | 3956  (77.2%) |  | 257  (5%) |  | 912  (17.8%) |  | 0  (0%) |  | 5018  (97.9%) |  | 107 (2.1%) |  | 0.834  (0.793–0.874) |  | 0.832  (0.761–0.903) |  | 0.836  (0.826–0.846) |
| Wave 6  (2012–13) | 4642 |  | 3525  (75.9%) |  | 267  (5.8%) |  | 850  (18.3%) |  | 0  (0%) |  | 4544  (97.9%) |  | 98 (2.1%) |  | 0.829  (0.786–0.872) |  | 0.827  (0.752–0.901) |  | 0.831  (0.820–0.842) |
| Wave 7  (2014–15) | 4067 |  | 3127  (76.9%) |  | 190  (4.7%) |  | 750  (18.4%) |  | 0  (0%) |  | 3963  (97.4%) |  | 104 (2.6%) |  | 0.805  (0.759–0.851) |  | 0.779  (0.699–0.859) |  | 0.831  (0.820–0.843) |
| Wave 8  (2016–17) | 3551 |  | 2764  (77.8%) |  | 125  (3.5%) |  | 662  (18.7%) |  | 0  (0%) |  | 3459  (97.4%) |  | 92 (2.6%) |  | 0.767  (0.714–0.820) |  | 0.707  (0.613–0.800) |  | 0.827  (0.815–0.840) |
| Wave 9  (2018–19) | 3033 |  | 2314  (76.3%) |  | 135  (4.4%) |  | 584  (19.3%) |  | 0  (0%) |  | 2939  (96.9%) |  | 94 (3.1%) |  | 0.785  (0.734–0.836) |  | 0.745  (0.657–0.833) |  | 0.825  (0.811–0.839) |
|  |  |  |  |  |  |  |  |  |  |  |  |  |  |  |  |  |  |  |  |

S Table 5. Comparison of self-reported dementia cases and Cluster 3 “Likely Dementia” cases in ELSA *AUC* Area under the Curve, *CI* confidence interval.

|  |  |  |  |  | **ELSA** |  |  |  |
| --- | --- | --- | --- | --- | --- | --- | --- | --- |
|  |  | **Main analysis** | | |  | **Sensitivity analysis** | | |
|  |  | **Model 1 (n=7840)** | | |  | **Model 2 (n=6784)** | | |
|  |  | **HR (95% CI)  (1 → 3)** |  | **HR (95% CI) (2 → 3)** |  | **HR (95% CI) (1 → 3)** |  | **HR (95% CI) (2 → 3)** |
|  |  |  |  |  |  |  |  |  |
| Low education |  | 1.89 (1.64-2.17) | | 1.19 (0.77-1.82) |  | 1.6 (1.37-1.87) |  | 1.07 (0.62-1.85) |
| Hearing loss |  | 1.75 (1.52-2.03) | | 1.35 (0.86-2.12) |  | 1.63 (1.39-1.91) | | 1.27 (0.7-2.29) |
| Hypertension |  | 1.42 (1.24-1.62) | | 1.64 (1.13-2.38) | | 1.39 (1.2-1.61) |  | 1.3 (0.78-2.16) |
| Drinking (> 21 units) |  | 0.6 (0.43-0.83) |  | 1.23 (0.68-2.24) |  | 0.79 (0.58-1.08) |  | 1 (0.37-2.7) |
| Smoking |  | 1.93 (1.6-2.33) |  | 1.63 (0.98-2.71) |  | 1.18 (1.79-2.64) | | 1.7 (0.87-3.33) |
| Depression |  | 2.03 (1.74-2.37) | | 2 (1.26-3.17) |  | 1.86 (1.57-2.19) | | 1.3 (0.69-2.42) |
| Social isolation |  | 1.61 (1.38-1.86) | | 1.52 (0.93-2.47) |  | 1.41 (1.2-1.65) |  | 1.61 (0.85-3.06) |
| Physical inactivity |  | 2.65 (2.27-3.1) |  | 2.69 (1.73-4.18) | | 1.74 (1.42-2.13) | | 2.02 (1.1-3.69) |
| Diabetes |  | 1.77 (1.38-2.26) | | 2.23 (1.26-3.95) | | 1.62 (1.24-2.13) | | 1.84 (0.87-3.9) |
| Obesity |  | 1.53 (1.32-1.77) | | 0.97 (0.63-1.48) |  | 1.62 (1.38-1.9) |  | 0.8 (0.44-1.46) |
| Pollution |  | NA |  | NA |  | NA |  | NA |
|  |  |  |  |  |  |  |  |  |

S Table 6. Multistate models for the transition to cluster 3 ("Likely Dementia") in ELSA Analyses using age as time-scale. All transitions were adjusted for sex. Transition towards the third cluster (“Likely Dementia”) was further adjusted for age and each risk factor individually. All risk factors were taken at baseline. Main analysis was based on a multistate model (Model 1). In sensitivity analysis, cases identified either at the first or the second wave were removed (Model 2). *HR* hazard ratio, *CI* confidence interval, *NA* not available
